# Imaging and biopsy of HIV-infected individuals undergoing analytic treatment interruption

**DOI:** 10.3389/fmed.2022.979756

**Published:** 2022-08-22

**Authors:** Chuen-Yen Lau, Matthew A. Adan, Jessica Earhart, Cassie Seamon, Thuy Nguyen, Ariana Savramis, Lindsey Adams, Mary-Elizabeth Zipparo, Erin Madeen, Kristi Huik, Zehava Grossman, Benjamin Chimukangara, Wahyu Nawang Wulan, Corina Millo, Avindra Nath, Bryan R. Smith, Ana M. Ortega-Villa, Michael Proschan, Bradford J. Wood, Dima A. Hammoud, Frank Maldarelli

**Affiliations:** ^1^HIV Dynamics and Replication Program, National Cancer Institute (NCI), National Institutes of Health (NIH), Bethesda, MD, United States; ^2^Critical Care Medicine Department, Clinical Center (CC), National Institutes of Health (NIH), Bethesda, MD, United States; ^3^HIV Dynamics and Replication Program, National Cancer Institute (NCI), National Institutes of Health (NIH), Frederick, MD, United States; ^4^PET Department, Clinical Center (CC), National Institutes of Health (NIH), Bethesda, MD, United States; ^5^Division of Neuroimmunology and Neurovirology, National Institute of Neurological Disorders and Stroke (NINDS), National Institutes of Health (NIH), Bethesda, MD, United States; ^6^Biostatistics Research Branch, National Institute of Allergy and Infectious Diseases (NIAID), National Institutes of Health (NIH), Bethesda, MD, United States; ^7^Interventional Radiology, Radiology and Imaging Sciences, Clinical Center (CC), National Institutes of Health (NIH), Bethesda, MD, United States; ^8^Radiology and Imaging Sciences, Clinical Center (CC), National Institutes of Health (NIH), Bethesda, MD, United States

**Keywords:** HIV, reservoir, treatment interruption, PET/CT, biopsy, clinical trial, HIV persistence

## Abstract

**Background:**

HIV persistence during antiretroviral therapy (ART) is the principal obstacle to cure. Lymphoid tissue is a compartment for HIV, but mechanisms of persistence during ART and viral rebound when ART is interrupted are inadequately understood. Metabolic activity in lymphoid tissue of patients on long-term ART is relatively low, and increases when ART is stopped. Increases in metabolic activity can be detected by ^18^F-fluorodeoxyglucose Positron Emission Tomography (FDG-PET) and may represent sites of HIV replication or immune activation in response to HIV replication.

**Methods:**

FDG-PET imaging will be used to identify areas of high and low metabolic uptake in lymphoid tissue of individuals undergoing long-term ART. Baseline tissue samples will be collected. Participants will then be randomized 1:1 to continue or interrupt ART *via* analytic treatment interruption (ATI). Image-guided biopsy will be repeated 10 days after ATI initiation. After ART restart criteria are met, image-guided biopsy will be repeated once viral suppression is re-achieved. Participants who continued ART will have a second FDG-PET and biopsies 12–16 weeks after the first. Genetic characteristics of HIV populations in areas of high and low FDG uptake will be assesed. Optional assessments of non-lymphoid anatomic compartments may be performed to evaluate HIV populations in distinct anatomic compartments.

**Anticipated results:**

We anticipate that PET standardized uptake values (SUV) will correlate with HIV viral RNA in biopsies of those regions and that lymph nodes with high SUV will have more viral RNA than those with low SUV within a patient. Individuals who undergo ATI are expected to have diverse viral populations upon viral rebound in lymphoid tissue. HIV populations in tissues may initially be phylogenetically diverse after ATI, with emergence of dominant viral species (clone) over time in plasma. Dominant viral species may represent the same HIV population seen before ATI.

**Discussion:**

This study will allow us to explore utility of PET for identification of HIV infected cells and determine whether high FDG uptake respresents areas of HIV replication, immune activation or both. We will also characterize HIV infected cell populations in different anatomic locations. The protocol will represent a platform to investigate persistence and agents that may target HIV populations.

**Study protocol registration:**

Identifier: NCT05419024.

## Introduction

HIV infection can be controlled, but not cured, by antiretroviral therapy (ART). The persistence of HIV-infected cells during ART is the principal reason for the inability to cure HIV with conventional therapy. Understanding persistence of HIV-infected cells during ART is critical; although HIV-infected cells are readily detectable during therapy in blood, their anatomic distribution remains uncertain, and new approaches to sample tissues and analyze HIV-infected cells from tissues are needed. The use of imaging technologies such as ^18^F-fluorodeoxyglucose Positron Emission Tomography (FDG-PET) to evaluate metabolic activity in studies of HIV infection dates back to the early years of the HIV epidemic. This was mostly for assessment of opportunistic infections, which were detected by increased metabolic activity. More recently, FDG-PET has been used to investigate the pathogenesis of HIV, and has demonstrated increased metabolic activity in lymph nodes of viremic untreated patients (1). Higher metabolic activity of spleen and lymph nodes also were found to correlate with plasma HIV viremia (1), while arterial inflammation correlated with soluble inflammatory markers in virally suppressed persons (2). The precise source of increased metabolic uptake is uncertain, and may be the result of HIV-infected cells that are metabolically active or the result of activation of immune cells directed against viral infection. The use of FDG-PET in the setting of long-term ART and ATI has the potential of visualizing the dynamics of viral rebound and lymphoid organ involvement, especially in the spleen, lymph nodes, and bone marrow.

### HIV persistence

Despite the availability of effective ART that suppresses viral replication, discontinuation of therapy results in prompt and rapid rebound in HIV viremia, and return of progressive immunodeficiency. HIV-infected individuals are obliged to lifelong ART for disease control. However, long-term ART is associated with complications of drug therapy, persistent immune activation, and emergence of drug resistance, particularly in the setting of non-adherence. New efforts to eradicate or control HIV infection are essential, but require a better understanding of viral persistence during therapy.

Untreated HIV infection proceeds with rapid virus replication, progressive CD4 depletion, and death from opportunistic infections or neoplastic diseases. Prior to initiating ART, HIV populations are relatively large (~10^4^-10^6^ infected cells transmitting HIV to uninfected cells per day) and genetically diverse (3). Infection results in cell death in the great majority (>99%) of infected T cells. A relatively small proportion of the entire CD4 cell compartment is infected, and the progressive CD4 lymphopenia characteristic of HIV-infected individuals is probably not the result of direct viral infection. Rather, progressive lymphopenia is likely due to immune mechanisms. HIV infection results in broad immune activation; mechanisms such as T-cell activation–induced cell death, T-cell exhaustion, and bystander effects of infected cells are likely responsible for progressive CD4 cell decline. There is active immunity to HIV, including soluble and cellular (CD4 and CD8) responses; despite these antiviral immune mechanisms, HIV persists.

During ART, the overwhelming majority of CD4 cells (~98%) are distributed in lymphoid tissue, including lymph nodes and spleen (4). Since these tissues are generally difficult to sample, much of our understanding of HIV infection has been derived studying plasma and peripheral blood mononuclear cells (PBMCs). As a result, sources of HIV persistence and anatomic locations of replication-competent HIV-infected cells remains uncertain.

Upon introduction of ART, new virus transmissions to uninfected cells are effectively blocked. Viral RNA levels in blood decline as new cells are not being infected and infected cells are eliminated, either by direct viral killing or by immune-based mechanisms to eliminate virus-infected cells. Viral RNA levels in plasma decline as infected cells are eliminated by multimodal decay kinetics. A sharp initial decline of 10- to 100-fold in the first 1–2 weeks of therapy corresponds to loss of cells with a relatively short half-life (1–2 days) (5). This initial decline is followed by a second phase decline, with a characteristic half-life of 2–3 weeks, corresponding to the loss of longer-lived cells, including macrophages and long-lived CD4+ T cells. Within 1–6 months, viral RNA levels decline to a level less than that quantifiable by commercial HIV RNA detection. Use of sensitive single-copy HIV RNA detection assays has revealed HIV RNA levels undergo a third phase decline with a half-life of ~39 weeks, likely corresponding to long-lived cell compartments. Analysis of individuals undergoing long-term ART has revealed persistent viremia with no further decay detected after 3 to 4 years (6).

HIV-infected cells persist during therapy and are readily detectable using sensitive techniques. Viral RNA levels decline a median of 17,000-fold after introduction of ART, but cell-associated viral DNA declines only 10- to 30-fold, suggesting a substantial population of remaining virus-infected cells (7, 8). These quantitative studies only measure the presence of HIV DNA and do not detect or quantify levels of replication-competent virus. Other detailed studies have consistently demonstrated that the level of replication-competent HIV is only a small fraction (1–3%) of the total number of infected cells, as demonstrated by Chun et al. and Bruner et al. (9–12). HIV-infected cells are present in peripheral blood as well as diverse anatomic compartments, including secondary lymphoid organs [lymph nodes, spleen, and gut-associated lymphoid tissue (GALT)], central nervous system (CNS), and renal tissue, all of which may represent viral sanctuaries.

Two potential mechanisms for the source of persistent low-level viremia have been proposed: (1) persistence of long-lived cells producing HIV in low-level constitutive fashion, or producing low-level viremia after transition from latent to active infection, and (2) continued cycles of HIV replication despite effective ART, because drugs are not completely effective or do not sufficiently penetrate all anatomic sites [gastrointestinal (GI) tract, CNS, renal tissue, etc.] with HIV-infected cells. Distinguishing between these two potential mechanisms is critical to the development of HIV eradication strategies. If ongoing cycles of HIV replication persist during therapy, then new and improved antiviral modalities are an essential component of curative strategies. Alternatively, if reservoirs of cells with replication-competent HIV are responsible for persistence, then new strategies to eliminate infected cells will be necessary.

In the past, studies have reported roles for both ongoing replication and for persistence of long-lived reservoirs of replication-competent HIV. Most recently, comparative analysis of HIV plasma viremia prior to and following ART revealed no evidence of molecular evolution of HIV populations, even after prolonged (>9 years) ART, suggesting little or no ongoing viral replication (8). Viral populations frequently include identical viral variants, suggestive of clonal expansion, and direct evidence of clonal expansion of HIV-infected cells has been demonstrated. Recent studies of replication-competent virus from clonally expanded CD4 T-cell lineages demonstrate that clonally expanded cells can harbor infectious HIV and that HIV-1 viremia during ART can originate from large T-cell clones (13, 14). Most infected cells, clonally expanded or not, contain defective proviruses, though the proportion of infectious proviruses that are clonally expanded is uncertain.

### Anatomic distribution of HIV-infected cells during ART

Anatomic locations and potential sanctuary sites of HIV infection have been investigated, but it is uncertain whether many of these sites represent a source of replication-competent HIV. Sensitive genetic techniques have been used to determine whether virus populations present in specific anatomic locations are compartmentalized, i.e., separate from the virus-producing cells present outside the anatomic location. A number of studies have suggested HIV compartmentalization in the genital tract, lymphoid tissue including GALT, and in the CNS. HIV infection of the CNS is established by infected cells trafficking across the blood-brain barrier to establish local infection. Virus enters the CNS within days of infection. Long-lived cells, including microglia, macrophages, and astrocytes in the brain can harbor the virus and also serve as a reservoir (15).

The role of the bone marrow in HIV remains poorly understood. Although bone marrow is an important source of CD4, CD8, and other immune cells in adults, it is unclear whether bone marrow represents a large site for persistence of HIV-infected cells. Differentiated bone marrow cells can become infected with HIV and infected marrow–derived monocytes may traffic HIV into the CNS. In turn, HIV proteins and associated cytokine changes can alter maturation of marrow cell lineages and cause apoptosis (16).

Another important HIV reservoir is the genital tract, which is often the site of primary infection. The uterine cervix may be an important reservoir, as rebound virus with ART cessation can be genetically identical to cervical sequences (17). HIV-1 populations in cervical and vaginal tissue may also have genetic features that differ from plasma virus, suggesting a degree of compartmentalization (18). Compartmentalization and clonal expansion in semen samples have also been identified in ART-naïve men (19). However, there are few analyses of HIV genetics and clonal expansion in blood and specific compartments in individuals suppressed for prolonged periods or after ATI. It is also important to understand these genital tract compartments because they represent sources of HIV transmission during treatment interruption.

### Imaging HIV-infected individuals

Traditional histologic, immunohistochemical, and molecular approaches to identify and characterize lymphoid tissue prior to and following introduction of ART are challenged by difficulties in routinely obtaining such tissues. Non-invasive imaging modalities have been explored to characterize HIV infection, persistence, and reactivation following ATI. PET is an imaging modality that is useful for localizing and quantifying sites of increased metabolic activity, which are generally over-represented in neoplastic or inflammatory/infectious processes. The radionuclide tracer F-18 FDG is an analog of glucose that is readily taken up into cells that are actively metabolizing glucose. FDG is typically transported by GLUT-1 and GLUT-3 (20) transporters, and undergoes phosphorylation by hexokinase to FDG-6-phosphate, however it does not undergo further metabolism, but accumulates in cells instead (21–23). FDG accumulates in tumor cells, macrophages, and other immune cells and granulation tissue. FDG-PET of healthy individuals demonstrates a physiologic biodistribution involving increased uptake in the brain and liver and excretion through the kidney; uptake in the heart is variable depending on the myocardial energy substrate (24). Studies of individuals with HIV infection have shown FDG-PET activity in the CNS, even in individuals with suppressed viremia (25, 26). Extensive studies of FDG-PET imaging in the setting of rebound viremia have not been performed to confirm that increased uptake represents local viral replication or to characterize contributing viral populations.

Activated lymphocytes and neutrophils also utilize high levels of glucose, comparable to that of malignant cells. FDG-PET has accordingly been explored for localizing sites of infection and in the evaluation of individuals with fever of unknown origin. In evaluation of individuals infected with HIV, FDG-PET is effective for distinguishing the discrete lesions of CNS lymphoma from cerebral toxoplasmosis, which may appear similar by computed tomography (CT) and magnetic resonance imaging (MRI). Using PET scans, the high metabolic activity of CNS lymphoma (FDG-avid) is typically distinguishable from the low metabolic activity appearance of toxoplasmosis (FDG-non-avid).

PET has also been used to identify sites of inflammation in various non-HIV conditions, such as vasculitides, osteomyelitis, and sarcoidosis (27).

Several disease stratification scoring systems based on FDG-PET have been developed, particularly for oncologic and rheumatologic conditions (28–32). The most commonly used semiquantitative measure is SUV (standard uptake value) representing the ratio of target tissue/lesion radioactivity concentration to the injected dose normalized to body weight/or lean body weight. In clinical practice, most studies report the SUV_max_, which is the maxium pixel value measured in the target lesion. However, the SUV_max_ does not represent the metabolic burden in the entire lesion since it is only derived from the highest pixel and does not provide information regarding the distribution of metabolic activity in the remaining lesion. More so, SUV_max_ is influenced by image noise and patient and imaging characteristics. Alternatively, SUV_peak_ has been used, representing the average pixel value within a fixed region of interest, usually of 1 cm^3^ volume.

A visual qualitative scoring system has also been found to be of high diagnostic accuracy. When comparing the lesion SUV with hepatic uptake, a fairly universal 4 grade system has been established: grade 0 = no evidence of FDG uptake in the lesion; grade 1= detectable uptake in the lesion of lower intensity than the liver; grade 2 = uptake in the lesion similar with liver uptake; grade 3= uptake in lesion above liver uptake. Alternatively, a ratio of the lesion uptake vs. the liver uptake can be derived.

Other quantitative measures have been studied and added value, particularly in treatment response assessment and disease prognosis: metabolic tumor volume (MTV) representing the tumor volume expressing a predermined threshold uptake as a percentage of the SUV_max_, and total lesion glycolysis (TLG) defined as the product of mean SUV and MTV (33). However, MTV and TLG require meticulous software-based analysis and are not used in clinical practice.

Less attention has been given to FDG based stratification systems for HIV and other infectious diseases. In our experience, providing a combined visual quantitative scoring, as described above, together with measurements of standard uptake values (SUV) in different target tissues/lesions, such as lymph nodes, spleen and bone marrow, will provide adequate information regarding metabolic activation status.

FDG-PET has previously been employed for investigations of HIV pathogenesis. In HIV-infected individuals not on ART, PET has detected areas of intense metabolic activity within secondary lymphoid tissue, especially lymph nodes that are not present in uninfected individuals. In quantitative studies, the degree of FDG uptake into lymphoid tissue is directly related to peripheral viral RNA levels, and inversely proportional to CD4 counts (34–36). The distribution of PET-positive nodes has been longitudinally evaluated after HIV infection, although extensive data are not available (37). Uptake into the spleen and GI tract, which contain substantial lymphoid tissue, has been described but uptake was inconsistently observed. HIV-infected cells or related activity may be diffusely distributed in large lymphoid organs, preventing clear visualization by standard PET approaches. Taken together, these data indicate ongoing immune activation during untreated HIV infection (38).

It has been assumed in studies of HIV infection that areas of metabolic activity correspond to active HIV replication and infected cells. However, HIV infection engenders broad immune activation, with levels of activation proportional to viral RNA levels in plasma. It is not certain that FDG uptake in lymphoid tissue corresponds to infected cells, broad immune activation, or both.

In contrast to PET studies of untreated HIV-infected patients showing marked FDG uptake, studies of virally suppressed HIV-infected individuals have revealed little or no FDG uptake in lymphoid tissues. No studies of individuals with persistent low-level viremia above clinical assay detection limits during ART have been performed. In our own experience, we evaluated a patient with squamous cell cancer and low-level HIV viremia due to a large population of HIV-infected cells who underwent PET to evaluate disease recurrence, and we did not detect any lymphoid uptake. Studies have investigated FDG uptake in animals and humans discontinuing ART. Schreiber-Stainthorp et al. found that ATI was associated with increased brain glucose metabolism in SIV-infected macaques (39). A study of SHIV infected macaques showed increased tissue FDG uptake preceded viral replication (34). In humans, Brust et al. noted strong uptake within 14 days of drug discontinuation, indicating rapid activation of metabolic activity after interrupting ART (35). Although FDG uptake may be detectable prior to increases in viral RNA levels, greater active uptake correlated with concurrent rapidly increasing plasma viral RNA levels. Combining PET scanning with CT localization is superior to PET alone (40–43), with improved resolution and increased specificity in detecting inflammation. However, specific studies in HIV pathogenesis have not been performed. In the present study, we will use state-of-the-art combined PET/CT localization in collaboration with interventional radiology colleagues who have pioneered the ability to identify and biopsy tissues of relatively small size.

Although metabolic activity has been detected in lymph nodes following ATI, it is not known what metabolically active sites represent. These may be primary sites of HIV replication or sites of early seeding of spreading virus; in contrast, they may not be sites of HIV replication at all, but simply sites of immune activation in response to viral rebound. Histology, immunohistochemistry, and *in situ* hybridization evaluation of nodal material from individuals undergoing ART were able to localize HIV DNA, but no such studies in individuals undergoing PET or ATI have been reported (44). A study of lymph node and GALT biopsies before and during ATI revealed a large number of viral sequences, likely representing recrudescent viremia from multiple sources (45). However, PET was not done in this study. Furthermore, not all nodes are FDG avid. During HIV infection, lymph nodes may become fibrotic with extensive collagen deposition, which may contribute to poor immune recovery (46–48). For patients continuing ART, changes in uptake would not be expected over a short period. Thus, imaging of these patients is not needed at the same frequency as in patients undergoing ATI.

Much can be learned about HIV persistence and reactivation by in-depth analysis of lymphoid tissue prior to and following ATI. Unfortunately, little clinical material from patients undergoing long-term ART or ATI has been available for study. The decrease in lymph node size that occurs during long-term effective ART prevents straightforward identification and facile surgical sampling for research studies. As a result of practical limitations, one of the most prominent sources of HIV persistence has not been extensively investigated in patients undergoing long-term ART. New approaches to study this key source of HIV infection are essential.

In the past several years, new imaging and interventional techniques have revolutionized identification, biopsy, and *in situ* treatment of diseased human tissues (49–52). The coregistration of PET, MRI, and CT facilitates accurate spatial identification of lesions; electromagnetic tracking (medical GPS) and optical tracking permit the positioning of biopsy instruments in real time (49, 53). The resulting precision has enabled targeted ablation of tumors such as paragangliomas, delicate vascular stent placement, and specific biopsy of smaller tissues, including adrenal and abnormal prostate tissue (54, 55). Using such techniques, lymphoid tissue has been routinely biopsied for clinical indications at NIH, and sampling of regressed lymph nodes after prolonged ART is well within the typical scope of image-guided biopsy.

### Analytic treatment interruption

Treatment interruptions have been studied over the last 15 years and have contributed significantly to understanding ART and HIV pathogenesis. Early studies demonstrated that cure from HIV is not possible with ART alone, and few patients maintain suppressed viral RNA levels following treatment interruption. Viral RNA levels are typically detectable within 2–4 weeks after ATI. During this early rebound period, viral RNA levels may rebound higher than viral set points prior to initial therapy, but with time off therapy, stable levels of viral RNA that are comparable to pre-therapy RNA are achieved. Shortly after treatment interruption, CD4 cell numbers may remain stable or decline slightly. New opportunistic infections are not an early consequence of treatment interruption (2–12 weeks post-interruption). The emergence of HIV drug resistance is not a typical consequence of discontinuing antiretroviral drugs (ARVs) with similar half-lives. In discontinuing regimens consisting of drugs with dissimilar half-lives [such as regimens containing non-nucleoside reverse transcriptase inhibitors (NNRTIs), which have half-lives more than 10-fold longer than nucleoside reverse transcriptase inhibitors (NRTIs) or protease inhibitors], there is a risk of emergence of NNRTI drug resistance due to the prolonged low level of NNRTI. Prolonged discontinuation from ART is associated with persistent viremia as well as progressive CD4 lymphopenia and disease progression. With the appropriate safeguards and patient counseling, ATI studies can be carried out without complications of drug resistance or HIV disease progression (56–64).

### Relevance to HIV cure

Characterization of HIV populations in different anatomic compartments contributing to the HIV reservoir will inform cure strategies. Understanding how those populations are activated or change in response to ATI may reveal unique opportunities for interventions that target specific reservoirs. As currently effective cure strategies are both life-threatening and not scalable, novel approaches are needed.

### Protocol plan

There are many gaps in understanding of HIV persistence and reactivation following ATI. The proposed study will use virologic, immunologic, genetic, and imaging approaches to investigate HIV persistence and kinetics of reactivation following short ART interruption. We will specifically evaluate the utility of FDG-PET for identifying changes in activity of potential HIV reservoirs in diverse anatomic locations. We will identify and biopsy FDG-avid and FDG-non-avid areas.

## Objectives and endpoints

Our primary objective is to assess the correlation of FDG-PET activity with changes in HIV RNA in lymphoid tissues before and after ATI. Secondary objectives will evaluate correlation of FDG-PET with HIV RNA in other compartments, as well as characterize changes in both DNA and RNA. Objectives and endpoints are summarized below in Table 1.

**Table 1:** Study objectives and endpoints.

**Objectives**	**Endpoints**	**Justification for endpoints**
**Primary**		
To evaluate if changes in glucose metabolism (as measured by FDG-PET SUV) correlate with changes in levels of HIV RNA in lymphoid tissue before and after ATI	Proportion of participants who have a 3-fold increase in HIV RNA levels in tissue sites identified by imaging as having increased SUV on FDG-PET as defined below	There are limited data on sensitivity of FDG-PET to detect HIV-infected cell populations during ATI. We will determine utility of this approach for investigating HIV persistence in people living with HIV
**Secondary**		
1. Characterize HIV populations (sequences) in sampled tissues, PBMCs, and plasma prior to and following ATI, and after treatment resumption 2. Assess relationship between changes in PET SUV and genetic characteristics (e.g., diversity, phylogenetics, and clonality) of HIV populations prior to, during, and after ATI 3. Assess relationship between changes in soluble and cellular immune parameters and imaging findings during viral rebound 4. Estimate replication competence of HIV variants from different anatomic compartments (sample tissues, PBMCs, plasma, semen, vaginal secretions, and CSF) 5. Compare kinetics of viral rebound after ATI with changes in immune activation markers	1. Levels of HIV DNA and integration site analysis to assess clonal distribution at different biopsy sites, semen, vaginal fluid, and PBMCs 2. Correlation between regional and overall change in PET SUV with HIV DNA and RNA sequencing characteristics pre-ATI to post-ATI 3. Cytokine and T-cell profiles during suppression and after ATI criteria for treatment resumption are met 4. HIV RNA and DNA sequence analyses for genetic studies and potential for replication competence 5. Correlation of HIV RNA levels and cytokine and T-cell profiles	HIV infection and viral replication are complex micro- and macro-level processes. Impact of ATI must be characterized at each of these levels for meaningful understanding of HIV persistence, and also to serve as a comparison point for future studies on this topic
**Tertiary/Exploratory**		
1. Evaluate interaction of host characteristics, HIV population characteristics, and immune profiles 2. Investigate HIV reactivation in other anatomic locations (CSF, semen, or vaginal secretions) in individuals who elect to undergo optional LP, or semen or vaginal secretion collection 3. Evaluate the relationship between FDG-PET SUV and immunologic activity 4. Investigate impact of ATI-associated changes in ART levels with changes in viral and immunologic characteristics	1. Correlations between HIV population markers, lymphocyte phenotype parameters, and participant characteristics 2. Comparative analysis of HIV DNA and RNA, levels of inflammatory markers, cytokine profiles, and antibody levels at different sites 3. Correlation between regional and overall change in PET SUV with changes in cytokine and T-cell profiles pre-ATI and post-ATI 4. Correlations of viral population changes, HIV DNA levels, HIV RNA levels, and immune markers with drug levels	Understanding the interaction between HIV populations and host characteristics is essential for devising eradication and cure strategies. Knowledge of adequacy of lymph node biopsy for virus characteristics and local immunologic milleu will inform future study design

## Methods and analysis

### Study design

**Figure 1:**
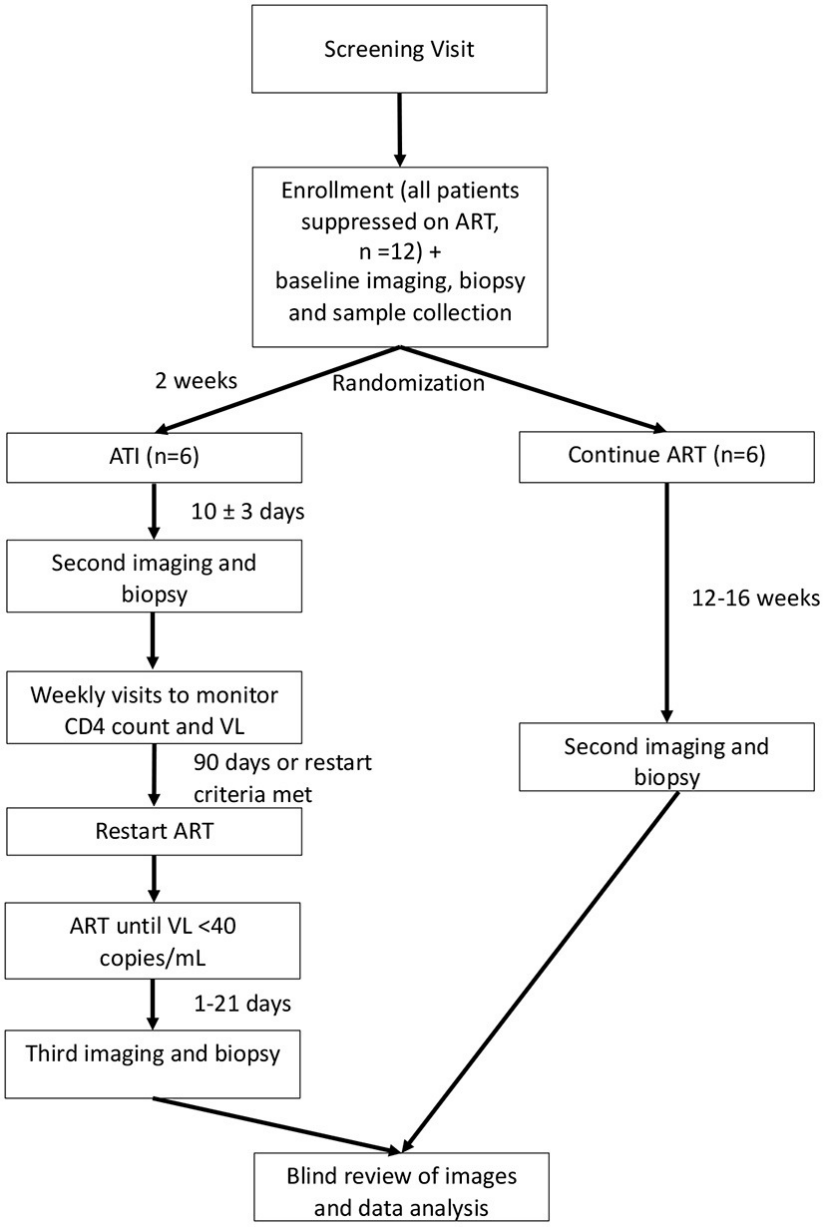
Study schema. ART, antiretroviral therapy; ATI, analytic treatment interruption; VL, viral load. Depicts the overall schema for participant randomization and study procedures. Participants will be randomized 1:1 to continue ART or undergo ATI. ATI participants will undergo a total of three PET/CTs with biopsy, while those who continue ART will receive two PET/CTs with biopsy.

This is a single-site randomized trial of the effects of ATI in six adult participants with HIV who are virally suppressed compared to six adult participants with HIV who are virally suppressed and who do not undergo ATI. Participants will be randomized 1:1 to either ATI or to continue their ART. Neither the participants nor the study team will be blinded, except for an imaging reader and biopsy reader, as described below. The study schema is shown in Figure 1. The schedule of events for participants randomized to ATI or to continue ART are shown in Supplementary Tables S2, S3.

#### Screening

Up to 50 individuals will be screened in order to identify 12 individuals who will be enrolled onto the study. Inclusion and exclusion criteria are described below. Those who are determined eligible will return for a baseline/first imaging visit.

#### Baseline/First imaging visit

At baseline, blood will be drawn *via* venipuncture for clinical and research evaluations, and participants will undergo whole body FDG PET/CT. Within the next 5 days, PBMCs will be collected by leukapheresis, and lymphoid tissue biopsies will be taken on the basis of imaging results. Approximately 2–5 biopsies will be performed after each PET scan. PET-avid (“hot”) and non-avid (“cold”) areas will be sampled. Lymph node biopsies will be guided by the combination of modalities most appropriate for each biopsy, which may include CT, ultrasound, and anatomic landmarks. Approximately 3 needle passes are typically performed, with the exact number guided by cytology feedback, intra-procedural factors, maintenance of low-risk, and size of target. Hot and cold nodes will be biopsied based on accessibility, with consideration for prior biopsies and consistency in biopsy site across participants. Optional sample collections are bone marrow biopsy, CSF *via* LP, and vaginal fluid or semen samples (which may be collected at home for the participant's convenience). Research evaluations on the samples are described in Table 2.

**Table 2:** Biospecimen evaluations.

**Samples to be collected**	**Collection method**	**Study requirements**	**Analyses planned for samples (assuming sufficient material obtained)**
Peripheral whole blood	Venicpuncture	Required	• Human leukocyte antigen (HLA) testing • Collection of PBMCs • Characterization of HIV populations • Sequencing of HIV-1, including ARV resistance testing • Inflammatory markers/cytokine profiling • Soluble markers of immune response • ARV drug levels • Interferon gene variant analysis • Storage for future research
Peripheral leukocytes	Leukapharesis	Required	• T-cell repertoire • Characterization of HIV populations • Sequencing of HIV-1, including ARV resistance testing
Cerebrospinal fluid	Lumbar puncture	Optional	• Albumin • Quantitative amino acids • Glucose • IgG • Total protein • Cell count and differential • Blood • HIV quantitative PCR • HIV sequencing • Cytokine analysis • Storage for future research
Lymph node tissue and bone marrow	Needle biopsy for lymph nodes, biopsy and aspirate for bone marrow	Lymph node biopsy is required, bone marrow biopsy is optional	• *In situ* hybridization, including RNAscope and DNAscope • HIV-1 viral load. HIV RNA levels will be normalized to total number of CD4 cells, quantified using CCR5 • Cell populations, flow cytometry • Immunohistochemistry • Cytokine analysis • Interferon sensitivity of replication-competent HIV recovered • Soluble markers of immune response • ARV drug levels • Genetic testing, including HLA typing • Storage for future research
Semen/vaginal fluid	Patient self-collection	Optional	• HIV RNA and DNA levels • Storage for future research

#### ATI or continue ART phase

After the baseline imaging visit, participants will be randomized 1:1 to either continue their ART regimen or undergo ATI, to begin 2 weeks (±3 days) after baseline. This interim serves two purposes: (1) to give ATI participants time to recover so they can be biopsied from the same site again if indicated, and (2) for those on ATI who take NNRTIs, to switch to a protease inhibitor or an integrase inhibitor–based regimen to ensure that the washout period of ARV drugs is roughly equal. The study team will work with the participant's regular care team to adjust ART regimens as needed and oversee the ATI. If necessary to ensure a safe discontinuation of ART, the interval between baseline and ATI initiation may be lengthened up to 8 weeks. ATI participants will have a second set of imaging and biospecimen collection 7–13 days after starting ATI. A biopsy reviewer who is blinded to cohort assignment (ATI or continuing ART) will review the whole body PET/CT images and determine what sites to biopsy.

Participants on ATI will have weekly study visits to monitor HIV symptoms, viral load, and CD4 count. When the participant meets a restart criterion, including reaching 90 days of interruption even if no other criterion is met, they will resume their ART at the next weekly visit. This ART restart visit will include a research blood draw. As this blood draw is critical to the study goals, participants who do not return for their ART restart visit will be replaced. Participants who withdraw prior to achievement of ART resumption criteria will similarly be replaced. However, their data will be kept as it can be used in the PET/CT and biopsy timing analyses. They will be advised on resuming their ART at home and will be followed for safety.

#### Resume ART phase or continue ART phase

Participants who underwent ATI will continue weekly visits after ART restart, and will undergo a third and final set of imaging and sample collection within 21 days after achieving viral suppression (HIV RNA < 40 copies/mL) to facilitate understanding of what increased SUV represents (viral activity, generalized inflammation, or another process). The blinded biopsy reviewer will again determine what sites to biopsy on the basis of the whole body PET/CT images. Participants will also have the option to undergo additional lymph node biopsies on ART restart days 0, 5, and/or 10, if the lymph nodes are superficial and easily accessible for biopsy, as determined by the radiologist. Participation in this study for the ATI group ends after the third imaging visit. Supplementary Table S2 summarizes the schedule of events for ATI participants.

Participants who remained on ART will have a second and final set of imaging and lymph node biopsies at 12–16 weeks after the first, which is anticipated to correspond with the final imaging and sample collection of the ATI group. As with the ATI group, the blinded biopsy reviewer will determine what sites to biopsy on the basis of the whole body PET/CT images. Participation in this study ends after this visit. A third set of imaging and biopsies will not be pursued for those who remained on ART, because changes in PET or biopsy findings are unlikely and would be unnecessary procedures. Supplementary Table S3 summarizes the schedule of events for participants who continue ART.

After these initial 12 participants complete the study, an NIH Clinical Center (CC) staff radiologist on the study team will act as a blinded imaging reader to review the whole body PET/CT results and attempt to identify whether the participant continued their ART regimen or was on ATI. This is a different person from the blinded biopsy reviewer. The blinded imaging reviewer's success rate, and the data analysis described below, will determine if the study should be repeated to optimize timing of imaging and biopsy. A routine clinical read of the whole body PET/CT scans will also be performed by a CC staff radiologist unaffiliated with the protocol who is not blinded, and actionable results provided to the participant and/or care providers.

Initial data analysis will entail measurement of total HIV RNA by polymerase chain reaction (PCR) from lymph node biopsy specimens. Comparisons will be made between the ATI and continued-ART groups, as well as within each randomization group across time points. This analysis will be used to (1) determine if ATI participants vs. controls could be identified *via* PET/CT, and (2) determine if viral RNA in lymph nodes increased over the course of ATI. Based on the results of the analysis of this group, we may need to adjust the timing of the first PET/CT with biopsy after ATI; this change will be done through a protocol amendment. If it is determined after the first 12 participants that the timing of the first PET/CT with biopsy should be adjusted, according to criteria outlined under “PET/CT and Biopsy Timing Justification” (0–1 ATI participants has >3 fold increase RNA in a biopsy site OR none of the 6 ATI participants are correctly classified), then the study would be repeated with new participants and a modified schedule with the 2nd imaging and biopsy assessment moved to 14–17 days post-ATI. If all 6 have 3 fold tissue RNA increase, we will move the imaging and biopsy to 3–6 days post-ATI. The study would be repeated with the same number of participants. Data analysis would be repeated using the same criteria. The study may be repeated more than once, if needed. Additional repetitions would shift the imaging and biopsy by increments of 4 days.

Study visits will typically be done as outpatients, but participants may be admitted as inpatients at the discretion of the investigators.

### Study population

#### Inclusion criteria

Participants must meet all of the following criteria to be eligible for this study:

1. Aged ≥ 18 years.
2. HIV-1 infection documented using FDA-approved screening and confirmatory or supplemental assays in Centers for Disease Control and Prevention (CDC)–recommended testing strategies.
3. Established medical care outside NIH.
4. Able to provide informed consent.
5. Willing to allow samples to be stored for future research.
6. Willing to allow genetic testing.
7. Undergoing ART using recommended, alternative, or other regimens as defined by “Guidelines for the use of Antiretroviral Agents in HIV-1 infected adults and Adolescents” (65).
8. Viral RNA < 40 copies/mL plasma by conventional assay for at least 3 years [blips (transient increases within 6 weeks) (66) of <200 copies/mL are allowable when succeeding viral levels return to <40 copies/mL on subsequent testing].
9. CD4 cell count ≥350 cell/μL.
10. Willing to interrupt ART for up to 90 days.
11. Willing to use a barrier method of contraception such as condoms or dams when engaging in sexual activity, or remain abstinent during ATI and after re-initiating ART until viral re-suppression is achieved, to prevent pregnancy and transmission of HIV.

#### Exclusion criteria

Participants who meet any of the following criteria will be excluded from this study:

1. Active intercurrent illness or infection, including fever >38°C.
2. Known history of initiating ART during the first year of infection with HIV. Patients will be considered to have initiated ART within 1 year of infection as defined by documented screening/confirmatory seroconversion [positive testing within 1 year of non-reactive HIV enzyme-linked immunosorbent assay (ELISA)].
3. Pregnant.
4. Breastfeeding.
5. Currently undergoing therapy with drugs that, in the judgment of the investigators, may interfere with biodistribution of FDG, including prednisolone, valproate, carbamazepine, phenytoin, phenobarbital, and catecholamines.
6. Undergoing ART that is incompatible with an ATI.
7. Has undergone PET/CT within the last 6 months.
8. History of poorly controlled diabetes that, in the judgment of the investigators, would prevent completion of PET/CT scan.
9. Vaccination within the previous 4 weeks.
10. History of ATI within the past 1 year.
11. Has comorbid illness for that, in the judgment of the investigators, an ATI will represent elevated risk.
12. Active opportunistic infection as defined by the Guidelines for the Prevention and Treatment of Opportunistic Infections in Adults and Adolescents with HIV (67).
13. Significant active substance abuse or psychiatric illness that may, in the judgment of the investigator, interfere with study visits or procedures.
14. Allergy to planned anesthetic agents that are expected to be used. For local anesthetics, this is lidocaine. For sedation, this is midazolam and fentanyl.
15. Currently undergoing chronic systemic steroid therapy (corticosteroid nasal spray or inhaler and topical steroid use are acceptable).
16. Contraindication to use of IV contrast.
17. History of developing keloids.
18. Renal impairment: HIV-related kidney disease or estimated glomerular filtration rate (eGFR) CKD-EPI equation <60 mL/min/m^2^. For individuals undergoing therapy with cobicistat or integrase strand inhibitors (INSTIs), GFR may be estimated using cystatin C or creatinine.
19. Active or chronic hepatitis B virus infection, with detectable hepatitis B surface antigen, hepatitis B virus DNA, or both.
20. Active hepatitis C virus infection, with detectable virus RNA.
21. History of HIV-associated dementia or progressive multifocal leukoencephalopathy.
22. Documented ARV drug resistance that, in the judgment of the investigator, would pose a risk of virologic failure should additional mutations develop during the study.
23. History of cardiovascular event or at high risk of an event (e.g., atherosclerotic cardiovascular disease score >20%) (https://tools.acc.org/ascvd-risk-estimator-plus/#!/calculate/estimate/).
24. History of AIDS-defining illness according to CDC criteria within the past 3 years (68).
25. Hepatic impairment: alanine transaminase >2.5 × the upper limit of normal or documented history of cirrhosis (69).
26. Any condition that, in the judgment of the investigator, contraindicates participation in this study.

### Study intervention

#### ATI

Participants randomized to ATI will halt their ART medications starting 2 weeks (±3 days) after the first imaging visit. This plan will be discussed with participants during the baseline visit. Patients will be contacted 1–3 days prior to ATI initiation. An interim history will be collected to ensure there are no new safety concerns. ATI may be delayed or canceled if there are new safety concerns. HIV plasma viral levels and CD4 counts will be monitored every week during the ATI phase. If a participant meets any of the ART restart criteria during the ATI phase, then they will discontinue ATI and restart ART. Participants who do not meet restart criteria will remain off ART and continue to be monitored weekly until they have been on ATI for 90 days, and then will restart ART.

#### Criteria to restart ART

Participants assigned to ATI will resume ART and continue the study if they meet any of the following criteria during the ATI phase:

- HIV RNA > 40 copies/mL and either a confirmed >30% decline in baseline CD4 cell count or an absolute CD4 count <300 cells/mL on 2 consecutive assesements within 2 weeks.
- A sustained (≥4 weeks) HIV RNA level of >1,000 copies/mL.
- Any HIV-related symptoms or acute retroviral syndrome presenting as fever, lymphadenopathy, sore throat, rash, myalgia/arthralgia, or diarrhea not already determined by physician examination to be related to something other than HIV.
- Any development of HIV-specific opportunistic infections, as listed in the DHHS guidelines (clinicalinfo.hiv.gov).
- ART is deemed medically necessary for non-HIV-related causes.
- Absolute HIV RNA >100,000 copies/mL.
- Participant becomes pregnant.
- At 90 days after starting ATI, any participant who has not met criteria to end the ATI will be instructed to restart ART.
- Participants who are in the ATI period who trigger a withdrawal criterion (Supplementary material S2) will also resume their pre-study ART regimen.

## Measures to minimize bias: Randomization and blinding

Randomization will be completed after study enrollment and will be 1:1 ATI to control (maintain ART). Image reviewers and biopsy reviewers will be blinded to randomization assignments until all 12 participants have completed the protocol and results have been analyzed as described in statistical considerations. The imaging reviewer is blinded as part of the determination of whether this study will be repeated. The biopsy reader is blinded to minimize bias in choosing which “hot” or “cold” nodes to biopsy. A Clinical Center (CC) staff radiologist who is not the blinded radiologist will review the PET/CT scans as they come in for clinical review and identification of sites to biopsy. As the ATI group will have three scans and the non-ATI group will have two scans, scans will be de-identified, and the first two scans will be used for discrimination of ATI vs. non-ATI to preserve blinding.

Randomization will be done by the Biostatistics Research Branch of the National Institute of Allergy and Infectious Diseases (NIAID). The randomization code will be maintained by the Biostatistics Research Branch on secure servers. The randomization code will be shared with all study staff except the blinded imaging and biopsy reviewers during the course of the study. Randomization will be performed *via* block randomization.

### Biospecimen evaluations

Biospecimen collection includes both required and optional samples. A summary of planned specimen collection and analyses to be conducted with these specimens is outlined in Table 2.

### Genetic/Genomic analysis

Somatic genetic testing may be conducted on blood, biopsy, and/or CSF samples. Genetic tests include HLA typing, interferon variant analysis, and mitochondrial DNA analysis.

The following may be done on samples collected under this study:

- In order to understand molecular evolution of HIV over time in infected individuals, HIV DNA and/or RNA will be sequenced. It will include both whole genome sequencing (9–10 kb) and HIV env sequencing (1.2 kb).
- Transcriptomic (sequencing from mRNA) analyses of cells.
- Limited genomic DNA sequencing to identify targeted polymorphisms. The limited genomic DNA sequencing may also include epigenetic analyses.
- Whole genome or whole exome sequencing will not be done under this protocol. All participants will be offered co-enrollment in the NIAID Centralized Sequencing Protocol (NIH IRB# 17-I-0122), but this is optional and not a condition of participation on this study.

## Design and statistical considerations

### Sample size justification

We will use a sample of size *n* = 12, with 6 participants randomized to the ATI group and 6 to the control group. A blinded PET/CT reader will attempt to correctly classify participants into their respective groups.

We determined the sample size of 12 participants (six on each arm) to allow for the blinded imaging reader to make an incorrect assignment and still obtain a significant *P*-value (*p* = 0.04) using a 1-tailed Fisher exact test and an alpha of 0.05. If we had used 10 participants (5 in each arm) instead of 12, then one incorrect assignment would yield a Fisher's exact test *P*-value of 0.103.

### Estimating the probability that an ATI participant has at least one productive hot spot

The probability p that an ATI participant has at least one hot spot that corresponds to a potential site of HIV replication or immune activation after ART discontinuation (we will use the term “productive hot spot”) will be estimated using a Bayesian procedure. Further details can be found in Supplementary material S1.

### Comparison of PET hot and cold biopsy samples for ATI participants

This will be restricted to ATI participants with abnormal hot spots; cold spots will be identified for comparison. HIV DNA values for hot and cold samples on each participant will be ranked and participant mean rank differences computed. A permutation test will be used to determine whether differences between cold and hot spots are beyond what would be explained by chance. Further details can be found in Supplementary material S1.

### PET/CT and biopsy timing justification

The timing of our interventions is based on current understanding of HIV rebound and imaging capability. If we do not find that analysis of FDG-PET is sufficiently sensitive to detect differences in metabolic activity, or we do not find increases in HIV RNA in tissues, the timing of imaging is likely too early and we will amend the protocol to extend the timing of the biopsy relative to the ATI start.

Conversely, if lymph node biopsies from all participants have a significant increase in HIV RNA after the first assessment, then the samples were likely collected too late and do not reflect the early stages of viral rebound. In this case, the study would be repeated with the first post-ATI assessment moved earlier. In some instances where consistency in biopsy site is not possible, different lymph nodes, preferably in the same region, will be biopsied across time points. Evidence suggests compartmentalization between different lymph nodes in the same individual does not occur (70).

After completing the analysis to examine data from the first 12 patients, the first PET/CT with biopsy will be moved from days 10 ± 3 post-ATI based on the following criteria:

a. Move later, to days 14 to 17 post-ATI

i). If 0/6 or 1/6 ATI patients have 3-fold increase in viral RNA
ii). If 0/6 ATI patients are recognized on PET/CT

b. Move earlier, to days 3 to 6 post-ATI

i). If 6/6 ATI patients have 3-fold increase in viral RNA

A 3-fold increase in HIV RNA is being used as the threshold because it represents an increase that is significantly different by single-copy assay.

## Discussion

HIV is controlled but not cured by combination antiretroviral therapy, and the mechanisms of HIV persistence remain poorly characterized. ATI studies have been useful in investigating the events in HIV rebound (71–77); many ATI studies analyze HIV present in plasma or in peripheral blood mononuclear cells. Most replication takes place in tissues, however, and HIV reactivation in tissues during ATI has not been well-studied. This protocol will allow us to explore utility of PET for identification of areas of high FDG uptake indictive of increased metabolic activity. By biopsying both FDG hot and FDG cold areas, we will be able to characterize these regions and investigate HIV replication during treatment interruption. One hypothesis is that PET SUV correlates with HIV viral RNA in biopsy of those regions and that lymph nodes with high SUV have higher viral RNA compared with areas with low SUV within a patient. If this is true, PET may be useful for identification of HIV infected cell populations, as well as monitoring response to cure strategies.

A randomized design was selected for this study to account for natural variation that might be seen in FDG uptake over time and to test the hypothesis that ATI engenders changes in FDG uptake not seen in patients who continue ART. Neither of these considerations could have been addressed without inclusion of the control ART continuation group. The possibility of doing only the ATI group, with each participant's baseline scan serving as their own control, was explored. Natural longitudinal variation in FDG uptake could, however, confound this approach. As Pfaehler and coworkers have reported, FDG PET uptake may be subject to variation from a number of potential sources (78, 79). Randomization to ATI vs. continuation of ART was necessary to optimize the balance of additional known and unknown confounders. The data generated, using rigorous uptake quantification, will also serve as an assessment of natural variation in FDG uptake over time, which will have general utility for FDG uptake studies.

A key feature of this protocol is that it will facilitate study of early events in HIV reactivation. Controlled ATI with frequent sample acquisition and targeted biopsies during the ATI will enable capture of data from very early timepoints, when reservoirs are expected to reactivate as suppression from ART wanes. In many current ATI studies, data on viral dynamics, genetic composition, immunologic responses, cell populations and anatomic localization may reveal biomarkers of persistence, and targets for intervention.

Individuals who undergo ATI are expected to have diverse viral populations upon viral rebound in lymphoid tissue. HIV populations in tissues may initially be phylogenetically diverse after ATI, with emergence of dominant viral species (clone) over time in plasma. Dominant viral species may represent the same HIV population seen before ATI. We will characterize reservoirs in different anatomic locations. Understanding local population dynamics will facilitate development of eradication and control strategies.

Our protocol design has built in flexibility to identify the optimal timeframe for studying reactivation events. Depending on the change in RNA or ability of the radiologist to classify ATI vs. non-ATI participants, schedules for imaging and biopsy may be shifted earlier or later. Establishment of optimal timing for reactivation studies, particularly with an imaging approach, will be useful in design of interventional studies evaluating HIV reservoirs and agents that may affect them (80–83).

## Ethics statement

The ethics of this study were evaluated by an NIH Scientific Review Committee and the Institutional Review Board (IRB), both of which agreed with the safety and scientific merit of this study. Ethical standards will be maintained in accordance with NIH and international policies. Participants will undergo informed consent when they join the study and will continue the conversation throughout their time on study. New information will be incorporated and resultant modifications, if any, will be discussed with participants. During the study intervention, participants will be closely monitored for safety signals, particularly those indicating the need to resume ART if assigned to the ATI arm. Study procedures will ensure that ATI is done safely and ethically, as outlined above. Incidental findings from validated studies will be reported back to participants and/or their medical provider. Furthermore, participants will be counseled on prevention of transmission in the setting of increasing viral load to protect others from becoming infected. Only volunteers who are already fully suppressed will be included in the study. Pregnant or breastfeeding women and children will be excluded. We have striven to include the broadest relevant population without taking undue risks. Although there is no benefit to individual participants, the larger HIV infected population will benefit from additional information about the reservoir. In order to maximize population benefit, specimens will be stored and data made available for future investigations. We anticipate completing this single site study over ~3 years. Study information and findings will be disseminated to stakeholders when they become available. Study information will be posted on Clinicaltrails.gov (NCT05419024) and shared with local providers and potential participants on other protocols. Findings will be published in peer reviewed journals after the data has been analyzed.

## Author contributions

C-YL, MA, AN, BS, and FM conceptualized the study. DH, CM, and BW provided guidance regarding radiologic aspects of the study. AO-V and MP completed stastical analyses required for protocol design. TN provided input on laboratory analyses. JE, CS, and AS provided clinical and administrative input. All authors provided intellectual input. All authors contributed to the article and approved the submitted version.

## Funding

This work has been funded by the Intramural Research Program, National Cancer Institute, Center for Cancer Research, and federal funds from the National Institute of Allergy and Infectious Diseases, NIH, the NIH Center for Interventional Oncology, and the Intramural Research Program of the National Institute of Biomedical Imaging and Bioengineering. Partial support comes from intramural NIH Grants Z1ACL040015 and 1ZIDBC011242. NIH may also have intellectual property in the field. A list of specific patents is available by request.

## Conflict of interest

Author BW would like to disclose the following: Licensed Patents/Royalties: Philips and NIH have a patent licensing agreement under which NIH receives royalties, a portion of which are then given to BW. NVIDIA and NIH have a licensing agreement. NIH and Canon have a licensing agreement. BW is Principal Investigator on the following Cooperative Research & Development Agreements (CRADAs), between NIH and industry: Philips (CRADA), Philips Research (CRADA), Celsion Corp (CRADA), BTG Biocompatibles/Boston Scientific (CRADA), Siemens (CRADA), NVIDIA (CRADA), XAct Robotics (CRADA). Negotiating CRADA with ProMaxo, Tempus, Galvanize, Theromics, Imactis, Varian. The following industry partners also support research in the Center for Interventional Oncology/Dr. Wood's lab via equipment, personnel, devices and/or drugs: 3T Technologies (devices), Exact Imaging (data), Angiodynamics (equipment), Astra Zeneca (pharmaceuticals, NCI CRADA), ArciTrax (devices and equipment), Imactis (Equipment), Johnson and Johnson (equipment), Medtronic (equipment), Promaxo (equipment & personnel), Theromics (Supplies), Profound (equipment and supplies), and QT Imaging (equipment and supplies). The remaining authors declare that the research was conducted in the absence of any commercial or financial relationships that could be construed as a potential conflict of interest.

## Publisher's note

All claims expressed in this article are solely those of the authors and do not necessarily represent those of their affiliated organizations, or those of the publisher, the editors and the reviewers. Any product that may be evaluated in this article, or claim that may be made by its manufacturer, is not guaranteed or endorsed by the publisher.

## Author disclaimer

The content of this publication does not necessarily reflect the views or policies of the Department of Health and Human Services, nor does mention of trade names, commercial products, or organizations imply endorsement by the U.S. Government.
