# Supplementary material for: Imaging and biopsy of HIV-infected individuals undergoing analytic treatment interruption
Source: Front Med (Lausanne). 2022 Aug 22;9:979756. doi: 10.3389/fmed.2022.979756 (PMC9441850; doi:10.3389/fmed.2022.979756)
Supplement: Supplementary file 1 [file Data_Sheet_1.docx]

# SUPPLEMENT 1: STATISTICAL CONSIDERATIONS

## Estimating the Probability that an ATI Participant Has at Least One Productive Hot Spot

We will estimate the probability p that an ATI participant has at least one hot spot that corresponds to a potential site of HIV replication or immune activation after ART discontinuation (we will use the term “productive hot spot”) using a Bayesian procedure described below. In this exploratory study in which a preliminary sample is used to compute the ultimate sample size, Bayesian methodology is appealing because inferences are based on the posterior (ie, updated) distribution of p given the data, and this posterior distribution is unaffected by adaptively computed sample sizes. Classical statistical approaches based on type 1 error rate do not have this advantage.

The Bayesian method quantifies prior opinion about p into a prior distribution, which is updated to a posterior distribution once data are observed. Our prior distribution is beta (1,1), equivalent to the uniform distribution on (0,1). In other words, the probability p is equally likely to be anywhere between 0 and 1. If we observe n people, x of whom have a productive hot spot, then the posterior distribution of p given the data is beta (1+x,1+n−x). We will use this posterior distribution to construct a credible interval (A,B) of likely values of p as follows. We determine A, such that the posterior probability that p is less than A is 0.025, and B such that the posterior probability that p exceeds B is 0.025. Then the posterior probability that p lies in the credible interval (A,B) is 0.95.

## Comparison of PET Hot and Cold Biopsy Samples for ATI Participants

For each ATI participant for whom there are abnormal hot spots, we will identify cold spots for comparison. We will use automated image analysis software to determine SUV, which will minimize bias during analysis. Lymph nodes with the greatest increase in standardized uptake value (SUV) will be prioritized for analysis.[^69-72^](#_ENREF_69) We will rank HIV DNA values for cold and hot samples on each ATI participant, and compute the difference Y_i_ between the mean rank for hot spots and the mean rank for cold spots for each participant. We then average the Y_i_ across people. To determine whether differences between hot and cold spots are beyond what would be explained by chance, we will conduct the following permutation test:

1. For participant i, flip a coin to generate either the actual difference Y_i_ or its mirror image −Y_i_.
2. Compute the average across people of the values generated in step 1.
3. Compute a two-tailed P value as the proportion of permuted datasets such that the average difference across people is at least as extreme as the actual average difference.

This permutation test is based on the idea that the difference in mean ranks follows a distribution that is symmetric about 0 if the null hypothesis is true. Therefore, when we condition on the absolute value of this difference, Y_i_ and −Y_i_ are equally likely. This test is akin to a paired t-test, but it is valid even if the data are not normally distributed.

The same approach described above for DNA will be repeated for each of the other secondary endpoints.

**SUPPLEMENT 2: WITHDRAWAL CRITERIA**

## Participant Discontinuation/Withdrawal from the Study

Participants are free to withdraw from participation in the study at any time upon request.

An individual participant will be withdrawn for any of the following:

- An individual participant’s decision. (The investigator should attempt to determine the reason for the participant’s decision.)
- Non-compliance with study procedures to the extent that it is potentially harmful to the participant or to the integrity of the study data.
- The participant loses the ability to provide ongoing informed consent.
- The participant does not have access to medical care outside the NIH.
- The participant becomes pregnant.
- The participant has evidence of a new sexually transmitted infection (STI). Participants will be monitored via discussion of sexual behavior and relevant history of STIs during all protocol visits. Evidence suggestive of STI will be investigated with targeted testing. If a new STI is documented, the participant will be discontinued from participation in study procedures, treated for STI, followed until therapy is completed, then formally withdrawn from the study.
- The investigator determines that continued participation in the study would not be in the best interest of the participant.

**SUPPLEMENT 3:** **ADVERSE EVENTS AND SERIOUS ADVERSE EVENTS**

### Definition of Adverse Event

An AE is any untoward medical occurrence in a human research participant, including any abnormal sign (for example, abnormal physical exam or laboratory finding), symptom, or disease, temporally associated with the individual’s participation in research, whether or not considered related to the individual’s participation in the research.

### Definition of Serious Adverse Events (SAE)

An SAE is any AE that:

- Results in death;
- Is life-threatening (places the participant at immediate risk of death from the event as it occurred);
- Results in inpatient hospitalization or prolongation of existing hospitalization;
- Results in a persistent or significant disability/incapacity;
- Results in a congenital anomaly/birth defect; OR
- Based upon appropriate medical judgment, may jeopardize the participant’s health and may require medical or surgical intervention to prevent one of the other outcomes listed in this definition (examples of such events include allergic bronchospasm requiring intensive treatment in the emergency room or at home, blood dyscrasias or convulsions that do not result in inpatient hospitalization, or the development of drug dependency or drug abuse).

### Classification of an Adverse Event

#### Specification of Safety Parameters

Participants may have very complicated and involved underlying etiologies at baseline. Therefore, only AEs related to the research procedures (including ATI) and not the participant’s underlying disease state will be followed in this protocol.

#### Severity of Event

The investigators will grade the severity of each AE according to the “Division of AIDS (DAIDS) Table for Grading the Severity of Adult and Pediatric Adverse Events” Version 2.1, July 2017, which can be found at <https://rsc.niaid.nih.gov/sites/default/files/daidsgradingcorrectedv21.pdf>.

Events that are NOT gradable using the above specified table will be graded as follows:

Mild = grade 1

Moderate = grade 2

Severe = grade 3

Potentially life threatening = grade 4

Death = grade 5

Some Grade 1 lab parameters on the DAIDS Toxicity Table fall within the NIH lab reference range for normal values. These normal values will not be reported as Grade 1 AEs.

#### Relationship to Research

All AEs must have their relationship to study intervention assessed by the investigator who examines and evaluates the participant based on temporal relationship and their clinical judgment. The degree of certainty about causality will be graded using the categories below. In a clinical trial, the study product must always be suspect.

**Definitely Related** – There is clear evidence to suggest a causal relationship, and other possible contributing factors can be ruled out. The clinical event, including an abnormal laboratory test result, occurs in a plausible time relationship to study intervention administration and cannot be explained by concurrent disease or other drugs or chemicals. The response to withdrawal of the study intervention (dechallenge) should be clinically plausible. The event must be pharmacologically or phenomenologically definitive, with use of a satisfactory rechallenge procedure if necessary.

**Probably Related** – There is evidence to suggest a causal relationship, and the influence of other factors is unlikely. The clinical event, including an abnormal laboratory test result, occurs within a reasonable time after administration of the study intervention, is unlikely to be attributed to concurrent disease or other drugs or chemicals, and follows a clinically reasonable response on withdrawal (dechallenge). Rechallenge information is not required to fulfill this definition.

**Potentially Related** – There is some evidence to suggest a causal relationship (eg, the event occurred within a reasonable time after administration of the trial medication). However, other factors may have contributed to the event (eg, the participant’s clinical condition, other concomitant events). Although an AE may rate only as “possibly related” soon after discovery, it can be flagged as requiring more information and later be upgraded to “probably related” or “definitely related”, as appropriate.

**Unlikely to be related** – A clinical event, including an abnormal laboratory test result, whose temporal relationship to study intervention administration makes a causal relationship improbable (eg, the event did not occur within a reasonable time after administration of the study intervention) and in which other drugs or chemicals or underlying disease provides plausible explanations (eg, the participant’s clinical condition, other concomitant treatments).

**Not Related** – The AE is completely independent of study intervention administration, and/or evidence exists that the event is definitely related to another etiology. There must be an alternative, definitive etiology documented by the clinician.

#### Expectedness

The investigator will be responsible for determining whether an AE is expected or unexpected. An AE will be considered unexpected if the nature, severity, or frequency of the event is not consistent with the risk information previously described for the research procedure.

### Time Period and Frequency for Event Assessment and Follow-Up

At each contact with the participant, information regarding AEs will be elicited by appropriate questioning and examinations. All events, both expected/unexpected and related/unrelated will be recorded on a source document. Source documents will include progress notes, laboratory reports, consult notes, phone call summaries, survey tools, and data collection tools. Source documents will be reviewed in a timely manner by the research team. All reportable AEs that are identified will be recorded in the NIAID electronic health record (CRIMSON). The start date, the stop date, the severity of each reportable event, and the principal investigator’s judgment of the AE’s relationship and expectedness to the research will also be recorded in CRIMSON.

AEs that have not resolved by the end of the follow-up period will be followed until final outcome is known. If it is not possible to obtain a final outcome for an AE (eg, the participant is lost to follow-up), the reason a final outcome could not be obtained will be recorded by the investigator in CRIMSON.

### AE Reporting

The principal investigator and/or a trained member of the study team will be responsible for conducting an evaluation of all AEs and shall report the results of such evaluation to the NIH IRB as per NIH HRPP Policy 801.

### SAE Reporting

SAEs will be reported to the NIH IRB per NIH HRPP Policy 801.

### Pregnancy

Although pregnancy itself is not an AE, events occurring during pregnancy, delivery, or in the neonate (eg, congenital anomaly/birth defect) may be AEs or SAEs.

In the event of pregnancy, the following steps will be taken:

If participant was in the ATI phase, then restart ART and withdraw from study.

Report to the IRB and safety monitoring committee.

Advise research participant to notify the obstetrician of study participation and study agent exposure.

## Unanticipated Problems

### Definition of Unanticipated Problems (UP)

Any incident, experience, or outcome that meets **all** of the following criteria:

- Unexpected in terms of nature, severity, or frequency given (a) the research procedures that are described in the protocol-related documents, such as the IRB-approved research protocol and informed consent document; and (b) the characteristics of the participant population being studied; and
- Related or possibly related to participation in the research (“possibly related” means there is a reasonable possibility that the incident, experience, or outcome may have been caused by the procedures involved in the research); and
- Suggests that the research places participants or others (which many include research staff, family members or other individuals not directly participating in the research) at a greater risk of harm (including physical, psychological, economic, or social harm) than was previously known or expected.

### UP Reporting

The investigator will report UPs to the NIH IRB as per HRPP Policy 801.

## Reporting Procedures

### Reporting to the NIH IRB

UPs, non-compliance, and other reportable events will be reported to the NIH IRB according to HRPP Policy 801.

### Reporting to the NIAID Clinical Director

The principal investigator will report UPs, major protocol deviations, and deaths to the NIAID Clinical Director according to institutional timelines.

Supplementary Table 1: Abbreviations

| AE | Adverse event |
| --- | --- |
| AIDS | Acquired immunodeficiency syndrome |
| ARV | Antiretroviral (drug) |
| ART | Antiretroviral therapy |
| ATI | Analytic treatment interruption |
| CBC/diff | Complete blood count with differential |
| CC | Clinical Center |
| CDC | Centers for Disease Control and Prevention |
| CFR | Code of Federal Regulations |
| CNS | Central nervous system |
| CRADA | Cooperative Research and Development Agreement |
| CRIMSON | Clinical Research Information Management System of the NIAID |
| CSF | Cerebrospinal fluid |
| CT | Computed tomography |
| DNA | Deoxyribonucleic acid |
| eGFR | Estimated glomerular filtration rate |
| ELISA | Enzyme-linked immunosorbent assay |
| FDA | Food and Drug Administration |
| FDG | ^18^F fluorodeoxyglucose |
| GALT | Gut-associated lymphoid tissue |
| GCP | Good Clinical Practice |
| GI | Gastrointestinal |
| HIV | Human immunodeficiency virus |
| HLA | Human leukocyte antigen |
| HRPP | Human Research Protection Program |
| ICH | International Council for Harmonisation of Technical Requirements for Pharmaceuticals for Human Use |
| IRB | Institutional review board |
| IV | Intravenous(ly) |
| LP | Lumbar puncture |
| MRI | Magnetic resonance imaging |
| NCI | National Cancer Institute |
| NIAID | National Institute of Allergy and Infectious Diseases |
| NIH | National Institutes of Health |
| NNRTI | Non‑nucleoside reverse transcriptase inhibitors |
| NRTI | Nucleoside reverse transcriptase inhibitor |
| OCRPRO | Office of Clinical Research Policy and Regulatory Operations |
| PBMC | Peripheral blood mononuclear cell |
| PCR | Polymerase chain reaction |
| PET | Positron emission tomography |
| QA | Quality assurance |
| QC | Quality control |
| RNA | Ribonucleic acid |
| SAE | Serious adverse event |
| SMC | Safety monitoring committee |
| SOA | Schedule of activities |
| SUV | Standard uptake value |
| UP | Unanticipated problem |
| US | United States |

Supplementary Table 2: Schedule of Activities for Participants Randomized to ATI

| **Evaluations** | **Screening**  **(Days −45 to −15)** | **Baseline/First Imaging Visit**  **(Day −14±3)^a,b^** | **ATI Phase** | | | **ART Resumption Phase** | | |
| --- | --- | --- | --- | --- | --- | --- | --- | --- |
|  |  |  | **Day 0** | **Second Imaging Visit**  **(Day 10±3)^a^** | **Weekly Visits (±3 Days)** | **ART Restart Visit^c^** | **Weekly Visits (±3 days)** | **Third Imaging Visit^d^** |
| Informed consent | X^e^ |  |  |  |  |  |  |  |
| Physical exam^f^ | X | X |  | X | X^g^ | X | X^g^ | X |
| Vital signs | X | X |  | X | X | X | X | X |
| Height | X |  |  |  |  |  |  |  |
| Weight | X | X |  | X | X | X | X | X |
| Medical history and medication review | X^h^ | X |  | X | X | X | X | X |
| AE assessment |  | X |  | X | X | X | X | X |
| Pregnancy test | X | X |  | X |  |  |  | X |
| Randomization |  | X |  |  |  |  |  |  |
| Start ATI |  |  | X |  |  |  |  |  |
| Resume ART |  |  |  |  |  | X |  |  |
| **Research procedures** |  |  |  |  |  |  |  |  |
| FDG-PET/CT scan |  | X |  | X |  |  |  | X |
| Lymph node biopsy^i^ |  | X^j^ |  | X^j^ |  | X^k^ | X^k^ | X^j^ |
| Leukapheresis |  | X^j^ |  | X^j^ |  |  |  | X^j^ |
| Optional LP^l^ |  | X^j^ |  | X^j^ |  |  |  | X^j^ |
| Optional bone marrow biopsy/aspirate |  | X^j^ |  | X^j^ |  |  |  | X^j^ |
| Optional semen sample/vaginal fluid^m^ |  | X^j^ |  | X^j^ |  |  |  | X^j^ |
| **Blood laboratory evaluations** |  |  |  |  |  |  |  |  |
| Hepatitis screen | 8.0 mL |  |  |  |  |  |  |  |
| CBC/diff | 3.0 mL | 3.0 mL |  | 3.0 mL | 3.0 mL | 3.0 mL | 3.0 mL | 3.0 mL |
| Lymphocyte flow cytometry^n^ | 6.0 mL | 6.0 mL |  | 6.0 mL | 6.0 mL | 6.0 mL | 6.0 mL | 6.0 mL |
| Acute care, hepatic, mineral panels | 3.5 mL | 3.5 mL |  | 3.5 mL | 3.5 mL^o^ | 3.5 mL | 3.5 mL^o^ | 3.5 mL |
| HIV RNA^p^ | 6.0 mL | 6.0 mL |  | 6.0 mL | 6.0 mL | 6.0 mL | 6.0 mL | 6.0 mL |
| HLA |  | 10.0 mL |  |  |  |  |  |  |
| Research analyses and storage^q^ |  | 120 mL |  | 80 mL |  | 120 mL |  | 120 mL |
| Maximum daily blood volume | 26.5 mL | 148.5 mL |  | 98.5 mL | 18.5 mL | 138.5 mL | 18.5 mL | 138.5 mL |
| AE, adverse event; ART, antiretroviral therapy; ARV, antiretroviral; ATI, analytic treatment interruption; CBC/diff, complete blood count with differential; CT, computed tomography; FDG, ^18^F fluorodeoxyglucose; HIV, human immunodeficiency virus; HLA, human leukocyte antigen; IFN, interferon; LP, lumbar puncture; PBMC, peripheral blood mononuclear cell; PET, positron emission tomography; RNA, ribonucleic acid; X, to be performed; [X], blood lab to be performed with a portion of the sample collected for the above lab (no additional blood required).  a This visit may be done over multiple days.  b Baseline visit will be conducted after confirmation of eligibility.  c The ART restart visit will be within 7 days of the participant triggering any of the protocol-specified ART restart criteria.  d Scheduled within 21 days of achieving viral suppression (HIV RNA<40 copies/mL).  e Informed consent will be obtained before any research procedures are done.  f Complete physical exam at screening, targeted exam at other study visits.  g To be done monthly.  h Limited review of existing medical records may be done before the participant signs the informed consent form.  i Lymph node biopsies may be guided by CT scan or ultrasound. CT-guided biopsies will not be used for the biopsy corresponding to the baseline scan.  j Collected within 5 days of FDG-PET/CT.  k Optional biopsies (up to 3 collected) at 0, 5, and 10 days after restarting ART if the lymph nodes are superficial and easily accessible for biopsy, as determined by the radiologist.  l LP may be guided by fluoroscopy.  m For their convenience, participants may be provided with collection kits to obtain these samples at home.  n Includes CD4+ T-cell count.  o These blood labs will be done once per month during the weekly visits for the ATI phase and ART resumption phase.  p HIV genotype will be obtained on all samples with HIV viral levels >1000 copies/mL.  q ARV levels will be checked in those required to switch ART regimens and others as needed. ARV levels will be repeated as needed to minimize risk of selecting for resistance. | | | | | | | | |

Supplementary Table 3: Schedule of Activities for Participants Randomized to Continue ART

| **Evaluations** | **Screening**  **(Days −45 to −15)** | **ART Continuation Phase** | |
| --- | --- | --- | --- |
|  |  | **Baseline/First Imaging Visit**  **(Day 0)** | **Second Imaging Visit**  **(Days 84 to 112)** |
| Informed consent | X^a^ |  |  |
| Physical exam^b^ | X | X | X |
| Vital signs | X | X | X |
| Height | X |  |  |
| Weight | X | X | X |
| Medical history and medication review | X^c^ | X | X |
| AE assessment |  | X | X |
| Pregnancy test | X | X | X |
| Randomization |  | X |  |
| **Research procedures** |  |  |  |
| FDG-PET/CT scan |  | X | X |
| Lymph node biopsy^d^ |  | X^e^ | X^e^ |
| Leukapheresis |  | X^e^ | X^e^ |
| Optional LP^f^ |  | X^e^ | X^e^ |
| Optional bone marrow biopsy/aspirate |  | X^e^ | X^e^ |
| Optional semen sample/vaginal fluid^g^ |  | X^e^ | X^e^ |
| **Blood laboratory evaluations** |  |  |  |
| Hepatitis screen | 8.0 mL |  |  |
| CBC/diff | 3.0 mL | 3.0 mL | 3.0 mL |
| Lymphocyte flow cytometry^h^ | 6.0 mL | 6.0 mL | 6.0 mL |
| Acute care, hepatic, mineral panels | 3.5 mL | 3.5 mL | 3.5 mL |
| HIV RNA^i^ | 6.0 mL | 6.0 mL | 6.0 mL |
| HLA |  | 10.0 mL |  |
| Research analyses and storage |  | 120 mL | 120 mL |
| Maximum daily blood volume | 26.5 mL | 148.5 mL | 138.5 mL |
| Abbreviations defined in Supplementary Table 1.  a Informed consent will be obtained before any research procedures are done.  b Complete physical exam at screening, targeted exam at other study visits.  c Limited review of existing medical records may be done before the participant signs the informed consent form.  d Lymph node biopsies may be guided by CT scan or ultrasound.  e Collected within 5 days of FDG-PET/CT.  f LP may be guided by fluoroscopy.  g For their convenience, participants may be provided with collection kits to obtain these samples at home.  h Includes CD4+ T-cell count.  i HIV genotype will be obtained on all samples with HIV viral levels >1000 copies/mL.  j ARV levels will be repeated as needed to minimize risk of selecting for resistance. | | | |
